# Generalized Proportional–Integral–Derivative Interpretation of a Class of Improved Two-Degree-of-Freedom Controllers

**DOI:** 10.3390/s26020466

**Published:** 2026-01-10

**Authors:** Wenfei Yu, Ping Lin, Shang Jiang, Xu Fang

**Affiliations:** 1 School of Control Science and Engineering, Dalian University of Technology, Dalian 116024, China; yuwenfei@mail.dlut.edu.cn (W.Y.); xufang@dlut.edu.cn (X.F.); 2 The Key Laboratory of Intelligent Control and Optimization for Industrial Equipment of Ministry of Education, Dalian University of Technology, Dalian 116024, China; 3 The Department of Missile and Naval Gun, Dalian Naval Academy, Dalian 116024, China

**Keywords:** generalized PID interpretation, improved linear ADRC, first-order linear ADRC, the second-order linear ADRC

## Abstract

In the framework of the traditional active disturbance rejection controller (ADRC), the state error feedback utilizes estimated values from the extended state observer, which may introduce phase lag. Therefore, academic researchers have proposed a modified version called the improved linear ADRC that employs output values from the plant for state error feedback except the output value from the extended state observer. However, there is limited literature exploring the relationship between traditional linear ADRC and improved linear ADRC. To address this gap, this article establishes mathmatical relationship between traditional linear ADRC and improved linear ADRC from the generalized PID control perspective, highlighting their distinctions in the frequency domain. Compared to the traditional ADRC, the improved ADRC incorporates differential terms and offers a novel approach to realize the generalized PID control via generalized PID interpretation. And to be more specific, the improved ADRC is a new way to realize the generalized PID control by three tuned parameters, and the number of parameters of the improved ADRC is fewer than that of the generalized PID control. From the time domain, numerical simulation results demonstrate that improved ADRC exhibits superior control performance by eliminating overshoot during set value tracking processes compared to the traditional ADRC. From the disturbance rejection simulations in direct current to direct current converter (DCDC), the improved ADRC can achieve better disturbance rejection performance than the traditional ADRC. The DC bus voltage drop values of the traditional ADRC are 55.8 V and 32 V; thus, the biggest voltage drop is 55.8 V, which is 7.44 times the the improved ADRC. The voltage rise of improved ADRC can be neglected compared to the voltage rise of the traditional ADRC. From the tracking performance perspective, the time to fully reach the reference value of the traditional ADRC is about 0.3 s, and the time to fully reach the reference value of the improved ADRC is about 0.15 s.

## 1. Introduction

The active disturbance rejection controller (ADRC), proposed by Prof. Han [1], has gained significant attention due to its exceptional disturbance rejection capability. To be more specific, the ADRC has tracking performance and disturbance rejection performance. For traditional proportional–integral controller, tracking performance and disturbance rejection performance are coupled. Once tracking performance is determined, disturbance rejection performance is also determined. For ADRC, tracking performance is dependent on the state error feedback control, and disturbance performance can de adjusted by the extended state observer. So, tracking performance and diturbance rejection performance are decoupled, which results in a two-degree-of-freedom controller. Thus, the revised version of the ADRC can be named improved two-degree-of-freedom controller.

ADRC has found applications in various industrial scenarios [2,3,4,5,6,7,8,9], including permanent magnet synchronous machines [10], induction motors [11], and grid-connected converters [12]. Given the stringent control requirements of these industrial scenarios, the robustness of ADRC plays a crucial role. Consequently, this research investigates approaches such as the linear-nonlinear switching ADRC [13] and the ADRC with integrator-extended state observer [14] to enhance the robustness of ADRC.

We present a control scheme that integrates linear and nonlinear switching mechanisms into an active disturbance rejection control (ADRC) framework, and the stability of the proposed scheme is analyzed using quantitative methods and compared to both linear and nonlinear ADRC approaches [13]. Additionally, the stability analysis of multiple input multiple output (MIMO) continuous systems based on linear/nonlinear switching ADRC is conducted in [15], building upon the work presented in [13]. The effectiveness of the proposed linear–nonlinear switching ADRC from [13] has been successfully extended to the context of permanent magnet synchronous motors in [16], demonstrating significant robustness. Regarding the stability assessment of this proposed approach, Lyapunov functions have been constructed as shown in both [17,18].

In the context of ADRC, a generalized integrator-extended state observer is proposed to effectively suppress extensive-scale rapidly varying sinusoidal disturbances in grid-connected converters scenario [14]. Building upon [14], Lin further proposes a generalized integrator-nonlinear extended state observer for accurate tracking and compensation of fast-varying sinusoidal disturbances, which has been validated using the dSPACE platform [19]. Additionally, a novel quasi-generalized integrator is proposed to suppress both periodic and aperiodic disturbances in the current loop of permanent magnet synchronous motors (PMSM) [20]. Furthermore, an innovative reduced-order vector resonant controller combined with generalized active disturbance rejection control is presented to effectively mitigate current disturbances containing periodic harmonics in PMSMs [21].

In the context of ADRC, the inclusion of state error feedback using output values from the plant can significantly enhance the robustness of revised ADRC compared to traditional ADRC by eliminating feedback phase lag. To ensure a fair comparison between traditional ADRC and improved ADRC, establishing a uniform scale is crucial, and this can be achieved through mathematical tools. However, researchers have not yet investigated the mathematical interrelation between traditional ADRC and improved ADRC with state error feedback using output values from the plant. Cao et al., in their literature review, provide an integrated explanation of active disturbance rejection control for electrical drives [22]. They employ a generalized proportional–integral–derivative (PID) controller to demonstrate the relationship between traditional linear ADRC and PI controller with reduced-order proportional–integral observer [23]. Furthermore, they analyze the mathematical relationship between high-order linear ADRC and cascade linear ADRC using generalized PID controller [24]. Inspired by these methods [22], we apply the generalized PID controller to analyze both the improved first-order linear ADRC and the improved second-order linear ADRC. And in [22,23,24], the traditional ADRC is analyzed by using the generalized PID control; thus, the relationship between the traditional ADRC and the improved ADRC is not determined.

The contribution of this article lies in the application of the generalized PID controller for analyzing the improved first-order linear ADRC and the improved second-order linear ADRC, thereby illustrating their distinctions through transfer function analysis. Specifically, compared to traditional linear ADRC, the improved ADRC incorporates differential terms in the perspective of the PID controller. To be more specific, this article establishes a mathmatical relationship between traditional linear ADRC and improved linear ADRC from the generalized PID control perspective, highlighting their distinctions in the frequency domain. Compared to the traditional ADRC, the improved ADRC incorporates differential terms and offers a novel approach to realize the generalized PID control via generalized PID interpretation. And to be more specific, the improved ADRC is a new way to realize the generalized PID control by three tuned parameters; the number of parameters of the improved ADRC is fewer than that of the generalized PID control. From the time domain, numerical simulation results demonstrate that improved ADRC exhibits superior control performance by eliminating overshoot during set value tracking processes compared to the traditional ADRC. From the disturbance rejection simulations in direct current to direct current converter (DCDC), the improved ADRC can obtain better disturbance rejection performance than the traditional ADRC.

The remainder of this paper is structured as follows. Section 2 presents the problem formulation. Section 3 presents the generalized PID interpretation of the improved first-order linear ADRC and the improved second-order linear ADRC. Section 4 gives the numerical simulation results. Conclusions and future work appear in Section 5.

## 2. Problem Formulation

**Figure 1:**
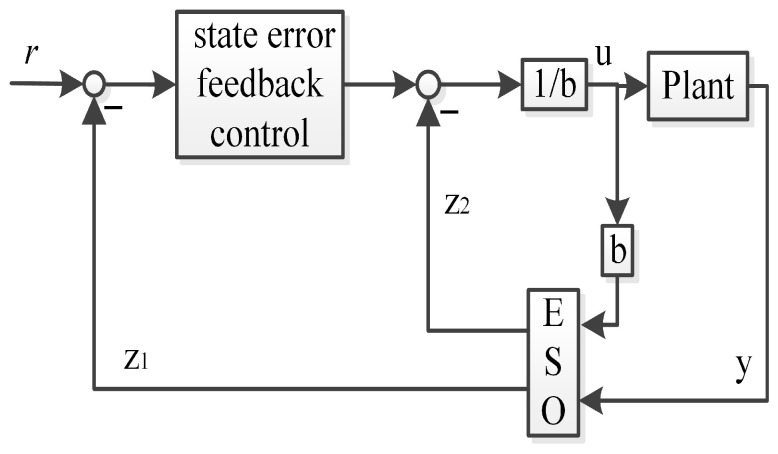
The traditional first-order linear ADRC.

The traditional framework of the linear ADRC includes the state error feedback control, the extended state observer (ESO), and the total disturbance compensation (Z2). The traditional first-order linear ADRC is presented in Figure 1.

**Figure 2:**
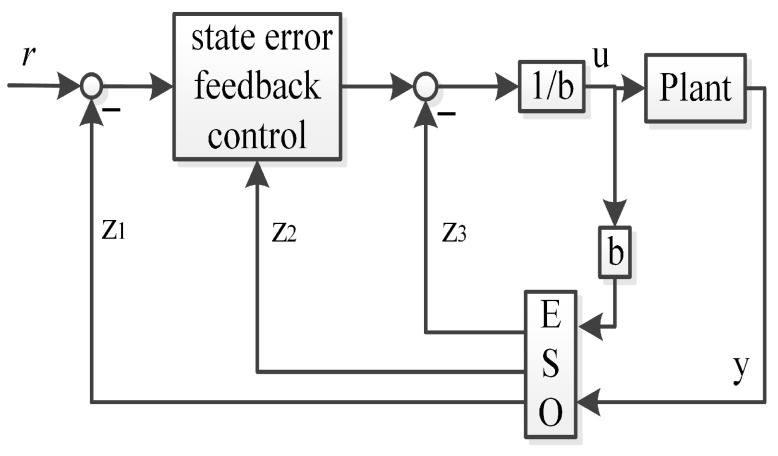
The traditional second-order linear ADRC.

The traditional second-order linear ADRC is illustrated in Figure 2.

**Figure 3:**
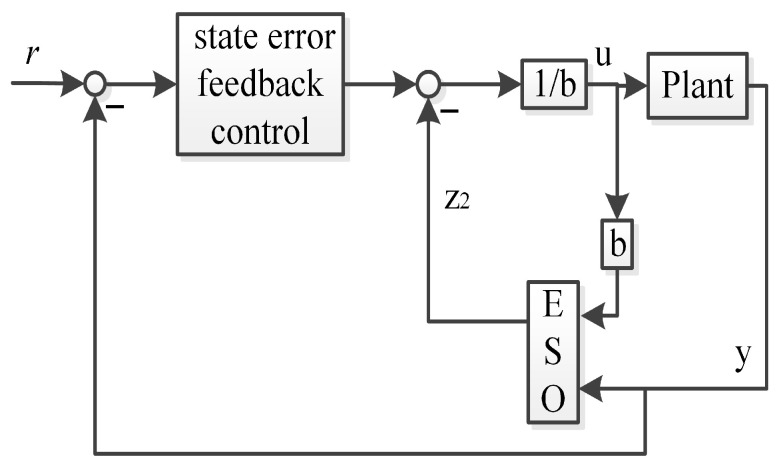
The improved first-order linear ADRC.

**Figure 4:**
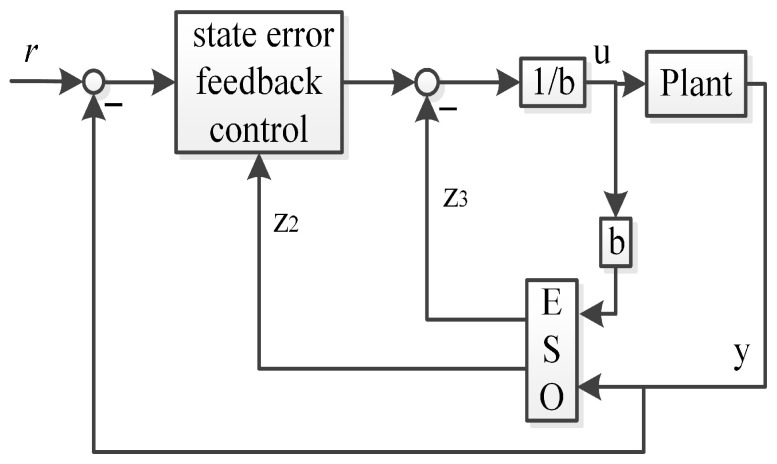
The improved second-order linear ADRC.

In order to mitigate the phase lag of the traditional linear ADRC in state error feedback control channel, the output values of the plant can be fed back to the state error feedback control channel. Thus, the improved first-order linear ADRC (IFLADRC) and the improved second-order linear ADRC (ISLADRC) are illustrated in Figure 3 and Figure 4, respectively.

The characteristics of the improved linear ADRC is that the state error feedback control uses the output value (y) from the plant instead of the values from the ESO (Z1), which mitigates the phase lag of the feedback values. We apply the generalized PID controller to analyze the IFLADRC and the ISLADRC, which illustrates the controller differences between the improved linear ADRC and the traditional linear ADRC by transfer function.

## 3. Main Results

The subsequent symbols are utilized consistently throughout this paper: $\beta_{11}$, $\beta_{12}$, $\beta_{21}$, $\beta_{22}$, and $\beta_{23}$ are gain coefficients of ESOs, $l_{11}$, $l_{21}$, and $l_{22}$ are coefficients of state error feedback control, and ${\bar{b}}_{0}$, ${\bar{b}}_{1}$ are estimated values of $b_{0}$, $b_{1}$, respectively.

### 3.1. The IFLADRC

**Lemma 1.**  *For some systems satisfying (1), (2), and (3), the IFLADRC can be interpreted as a PID controller in series with a filter.*

**Proof.**  It is assumed that the plant depicted in Figure 1 and Figure 3 can be characterized as follows:

(1) $$\begin{array}{ccc} {\dot{x}}_{1} & = & x_{2}+b_{1}u \end{array}$$

(2) $$\begin{array}{ccc} {\dot{x}}_{2} & = & f \end{array}$$

(3) $$\begin{array}{ccc} y & = & x_{1} \end{array}$$

The linear ESO and state error feedback control in the framework of traditional linear first-order ADRC can be illustrated as follows:

(4) $$\begin{array}{ccc} {\dot{z}}_{1} & = & \beta_{11}\left(y-z_{1}\right)+z_{2}+{\bar{b}}_{1}u \end{array}$$

(5) $$\begin{array}{ccc} {\dot{z}}_{2} & = & \beta_{12}\left(y-z_{1}\right) \end{array}$$

(6) $$\begin{array}{ccc} u & = & \frac{1}{{\bar{b}}_{1}}\left(l_{11}r-l_{11}z_{1}-z_{2}\right) \end{array}$$

where $z_{1}$ and $z_{2}$ are observed values of $x_{1}$ and $x_{2}$, respectively. Equations (4)–(6) are consistent with Figure 1.

The linear ESO and the improved state error feedback control in the framework of the improved linear first-order ADRC can be illustrated as follows:

(7) $$\begin{array}{ccc} {\dot{z}}_{1} & = & \beta_{11}\left(y-z_{1}\right)+z_{2}+{\bar{b}}_{1}u \end{array}$$

(8) $$\begin{array}{ccc} {\dot{z}}_{2} & = & \beta_{12}\left(y-z_{1}\right) \end{array}$$

(9) $$\begin{array}{ccc} u & = & \frac{1}{{\bar{b}}_{1}}\left(l_{11}r-l_{11}y-z_{2}\right) \end{array}$$

Here, $z_{1}$ and $z_{2}$ represent the estimated values of $x_{1}$ and $x_{2}$, respectively. Equations (7)–(9) are consistent with Figure 3.

We rewrite the linear ESO and the improved state error feedback control as follows:

(10) $$\begin{array}{ccc} {\dot{z}}_{1} & = & -\beta_{11}z_{1}+\left(\beta_{11}-l_{11}\right)y+l_{11}r \end{array}$$

(11) $$\begin{array}{ccc} {\dot{z}}_{2} & = & -\beta_{12}z_{1}+\beta_{12}y \end{array}$$

(12) $$\begin{array}{ccc} u & = & \frac{1}{{\bar{b}}_{1}}\left(l_{11}r-l_{11}y-z_{2}\right) \end{array}$$

We treat *y* and *r* as the input value and *u* as the output value. We can get the following transfer functions as follows:

(13) $$\frac{U(s)}{Y(s)}=\frac{-l_{11}s^{2}-\left(l_{11}\beta_{11}+\beta_{12}\right)s-\beta_{12}l_{11}}{{\bar{b}}_{1}s\left(s+\beta_{11}\right)}$$

(14) $$\frac{U(s)}{R(s)}=\frac{l_{11}\left(s^{2}+\beta_{11}s+\beta_{12}\right)}{{\bar{b}}_{1}s\left(s+\beta_{11}\right)}$$

By mathematical derivation, we can get

(15) $$C_{11}\left(s\right)=\frac{l_{11}s^{2}+\left(l_{11}\beta_{11}+\beta_{12}\right)s+\beta_{12}l_{11}}{{\bar{b}}_{1}s\left(s+\beta_{11}\right)}$$

(16) $$H_{11}\left(s\right)=\frac{l_{11}\left(s^{2}+\beta_{11}s+\beta_{12}\right)}{l_{11}s^{2}+\left(l_{11}\beta_{11}+\beta_{12}\right)s+\beta_{12}l_{11}}$$

We can rewrite $C_{11}\left(s\right)$ as follows:

(17) $$C_{11}\left(s\right)=\frac{l_{11}s^{2}+\left(l_{11}\beta_{11}+\beta_{12}\right)s+\beta_{12}l_{11}}{{\bar{b}}_{1}s\beta_{11}}\frac{\beta_{11}}{\left(s+\beta_{11}\right)}$$

By observing (17), we can get

$$k_{p11}=\frac{l_{11}\beta_{11}+\beta_{12}}{{\bar{b}}_{1}\beta_{11}}$$

$$k_{d11}=\frac{l_{11}}{{\bar{b}}_{1}\beta_{11}}$$

$$k_{i11}=\frac{l_{11}\beta_{12}}{{\bar{b}}_{1}\beta_{11}}$$

where $k_{p}$ represents the proportional gain, $k_{i}$ denotes the integral gain, and $k_{d}$ signifies the derivative gain. The proof of **Lemma 1** is finished. □

By observing (4)–(6), we can get

(18) $$C_{12}\left(s\right)=\left(k_{p12}+k_{i12}\frac{1}{s}\right)\frac{\beta_{11}l_{11}}{s+\beta_{11}+l_{11}}$$

where

$$k_{p12}=\frac{\beta_{11}l_{11}+\beta_{12}}{{\bar{b}}_{1}\left(\beta_{11}+l_{11}\right)}$$

$$k_{i12}=\frac{\beta_{12}l_{11}}{{\bar{b}}_{1}\left(\beta_{11}+l_{11}\right)}$$

Thus, the traditional first-order linear ADRC can be interpreted as a PI controller in series with a low-pass filter, which is also proved in [25].

(19) $$H_{12}\left(s\right)=\frac{l_{11}\left(s^{2}+\beta_{11}s+\beta_{12}\right)}{\left(\beta_{11}l_{11}+\beta_{12}\right)s+\beta_{12}l_{11}}$$

### 3.2. The Improved Second-Order Linear ADRC

**Lemma 2.**  *For some systems satisfying (20)–(23), the ISLADRC can be interpreted as a PIDD^2^ controller in series with a filter.*

**Proof.**  We assume that the Plant in Figure 2 and Figure 4 can be described as follows:

(20) $$\begin{array}{ccc} {\dot{x}}_{1} & = & x_{2} \end{array}$$

(21) $$\begin{array}{ccc} {\dot{x}}_{2} & = & x_{3}+b_{2}u \end{array}$$

(22) $$\begin{array}{ccc} {\dot{x}}_{3} & = & h \end{array}$$

(23) $$\begin{array}{ccc} y & = & x_{1} \end{array}$$

The linear ESO and state error feedback control in the framework of the traditional linear second-order ADRC can be illustrated as follows:

(24) $$\begin{array}{ccc} {\dot{z}}_{1} & = & \beta_{21}\left(y-z_{1}\right)+z_{2} \end{array}$$

(25) $$\begin{array}{ccc} {\dot{z}}_{2} & = & \beta_{22}\left(y-z_{1}\right)+z_{3}+{\bar{b}}_{2}u \end{array}$$

(26) $$\begin{array}{ccc} {\dot{z}}_{3} & = & \beta_{23}\left(y-z_{1}\right) \end{array}$$

(27) $$\begin{array}{ccc} u & = & \frac{1}{{\bar{b}}_{2}}\left(l_{22}r-l_{22}z_{1}-l_{21}z_{2}-z_{3}\right) \end{array}$$

where $z_{1}$, $z_{2}$, and $z_{3}$ are observed values of $x_{1}$, $x_{2}$, and $x_{3}$, respectively. Equations (24)–(27) are consistent with Figure 2.

The linear ESO and the improved state error feedback control in the framework of the ISLADRC can be illustrated as follows:

(28) $${\dot{z}}_{1}=\beta_{21}\left(y-z_{1}\right)+z_{2}$$

(29) $${\dot{z}}_{2}=\beta_{22}\left(y-z_{1}\right)+z_{3}+{\bar{b}}_{2}u$$

(30) $${\dot{z}}_{3}=\beta_{23}\left(y-z_{1}\right)$$

(31) $$u=\frac{1}{{\bar{b}}_{2}}\left(l_{22}r-l_{22}y-l_{21}z_{2}-z_{3}\right)$$

Equations (28)–(31) are consistent with Figure 4.

Substituting (31) into (29), we can get

(32) $$\begin{array}{ccc} {\dot{z}}_{1} & = & -\beta_{21}z_{1}+z_{2}+\beta_{21}y \end{array}$$

(33) $$\begin{array}{ccc} {\dot{z}}_{2} & = & -\beta_{22}z_{1}-l_{21}z_{2}+\left(\beta_{22}-l_{22}\right)y+l_{22}r \end{array}$$

(34) $$\begin{array}{ccc} {\dot{z}}_{3} & = & -\beta_{23}z_{1}+\beta_{23}y \end{array}$$

(35) $$\begin{array}{ccc} u & = & \frac{1}{{\bar{b}}_{2}}\left(l_{22}r-l_{22}y-l_{21}z_{2}-z_{3}\right) \end{array}$$

We treat *y* and *r* as the input value and *u* as the output value. We can get the following transfer functions as follows:

(36) $$\frac{U(s)}{Y(s)}=-\frac{l_{22}s^{3}+a_{1}s^{2}+a_{2}s+l_{22}\beta_{23}}{\left({\bar{b}}_{2}\left(\beta_{22}s+\beta_{21}s^{2}+l_{21}s^{2}+s^{3}+\beta_{21}l_{21}s\right)\right)}$$

(37) $$\frac{U(s)}{R(s)}=\frac{l_{22}\left(\beta_{22}s+\beta_{21}s^{2}+l_{21}s^{2}+s^{3}+\beta_{21}l_{21}s\right)}{\left({\bar{b}}_{2}\left(\beta_{22}s+\beta_{21}s^{2}+l_{21}s^{2}+s^{3}+\beta_{21}l_{21}s\right)\right)}$$

By mathematical derivation, we can get

(38) $$C_{21}\left(s\right)=\frac{l_{22}s^{3}+a_{1}s^{2}+a_{2}s+l_{22}\beta_{23}}{\left({\bar{b}}_{2}\left(\beta_{22}s+\beta_{21}s^{2}+l_{21}s^{2}+s^{3}+\beta_{21}l_{21}s\right)\right)}$$

(39) $$H_{21}\left(s\right)=\frac{l_{22}\left(\beta_{22}s+\beta_{21}s^{2}+l_{21}s^{2}+s^{3}+\beta_{21}l_{21}s\right)}{l_{22}s^{3}+a_{1}s^{2}+a_{2}s+l_{22}\beta_{23}}$$

where $a_{1}=\left(\beta_{21}l_{22}+\beta_{23}+2l_{21}l_{22}-l_{21}\beta_{22}\right)$, $a_{2}=\left(l_{22}\beta_{22}+l_{21}\beta_{23}+2l_{21}l_{22}\beta_{21}-2l_{21}\beta_{21}\beta_{22}\right)$.

Then, we rewrite $C_{21}\left(s\right)$

(40) $$C_{21}\left(s\right)=\left(k_{dd21}s^{2}+k_{d21}s+k_{p21}+k_{i21}\frac{1}{s}\right)F_{L21}$$

where

$$k_{dd21}=\frac{l_{22}}{{\bar{b}}_{2}\left(\beta_{21}l_{21}+\beta_{22}\right)}$$

$$k_{d21}=\frac{a_{1}}{{\bar{b}}_{2}\left(\beta_{21}l_{21}+\beta_{22}\right)}$$

$$k_{p21}=\frac{a_{2}}{{\bar{b}}_{2}\left(\beta_{21}l_{21}+\beta_{22}\right)}$$

$$k_{i21}=\frac{l_{22}\beta_{23}}{{\bar{b}}_{2}\left(\beta_{21}l_{21}+\beta_{22}\right)}$$

$$F_{L21}=\frac{\beta_{21}l_{21}+\beta_{22}}{\beta_{22}+\beta_{21}s+l_{21}s+s^{2}+\beta_{21}l_{21}}$$

By observing (40), we can determine that the improved second-order linear ADRC can be interpreted as a PIDD^2^ controller in series with a low-pass filter. The proof of **Lemma 2** is finished. □

By observing (24)–(27), we can get

(41) $$C_{22}\left(s\right)=\left(k_{d22}s+k_{p22}+k_{i22}\frac{1}{s}\right)F_{L22}$$

where

$$k_{p22}=\frac{\beta_{22}l_{22}+\beta_{23}l_{21}}{{\bar{b}}_{2}\left(\beta_{21}l_{21}+\beta_{22}+l_{22}\right)}$$

$$k_{i22}=\frac{\beta_{23}l_{22}}{{\bar{b}}_{2}\left(\beta_{21}l_{21}+\beta_{22}+l_{22}\right)}$$

$$k_{d22}=\frac{\beta_{21}l_{22}+\beta_{22}l_{21}+\beta_{23}}{{\bar{b}}_{2}\left(\beta_{21}l_{21}+\beta_{22}+l_{22}\right)}$$

$$F_{L22}=\frac{\beta_{21}l_{21}+\beta_{22}+l_{22}}{s^{2}+\left(\beta_{21}+l_{21}\right)s+\beta_{21}l_{21}+\beta_{22}+l_{22}}$$

(42) $$H_{22}\left(s\right)=\frac{l_{22}\left(s^{3}+\beta_{21}s^{2}+\beta_{22}s+\beta_{23}\right)}{H_{22m(s)}}$$

where

$$H_{22m(s)}=\left(\beta_{21}l_{22}+\beta_{22}l_{21}+\beta_{23}\right)s^{2}+\left(\beta_{22}l_{22}+\beta_{23}l_{21}\right)s+\beta_{23}l_{22}$$

Thus, the traditional second-order linear ADRC can be interpreted as a PID controller in series with a low-pass filter, which is also proved in [25].

(43) $$CL_{11}\left(s\right)=\frac{Y(s)}{R(s)}=\frac{H_{11}C_{11}P_{1}}{1+C_{11}P_{1}}$$

(44) $$CL_{12}\left(s\right)=\frac{Y(s)}{R(s)}=\frac{H_{12}C_{12}P_{1}}{1+C_{12}P_{1}}$$

(45) $$CL_{21}\left(s\right)=\frac{Y(s)}{R(s)}=\frac{H_{21}C_{21}P_{2}}{1+C_{21}P_{2}}$$

(46) $$CL_{22}\left(s\right)=\frac{Y(s)}{R(s)}=\frac{H_{22}C_{22}P_{2}}{1+C_{22}P_{2}}$$

(47) $$D_{11}\left(s\right)=\frac{Y(s)}{D_{2}\left(s\right)}=\frac{P_{1}}{1+C_{11}P_{1}}$$

(48) $$D_{12}\left(s\right)=\frac{Y(s)}{D_{2}\left(s\right)}=\frac{P_{1}}{1+C_{12}P_{1}}$$

(49) $$D_{21}\left(s\right)=\frac{Y(s)}{D_{2}\left(s\right)}=\frac{P_{2}}{1+C_{21}P_{2}}$$

(50) $$D_{22}\left(s\right)=\frac{Y(s)}{D_{2}\left(s\right)}=\frac{P_{2}}{1+C_{22}P_{2}}$$

### 3.3. The Discussions of the Improved First-Order and Second-Order Linear ADRC

By observing $C_{11}\left(s\right)$, the state error feedback control channel uses the output value y in the improved first-order linear active disturbance rejection controller (IFLADRC), different from the state error feedback control channel utilizing the estimated value z in the traditional first-order linear active disturbance rejection controller from the extended state observer. For the improved second-order linear active disturbance rejection controller (IFLADRC), $C_{21}\left(s\right)$, adopting the state error feedback control channel, uses the output value y, different from the state error feedback control channel using the estimated value z from the extended state observer in the framework of traditional second-order linear active disturbance rejection controller.

**Figure 5:**
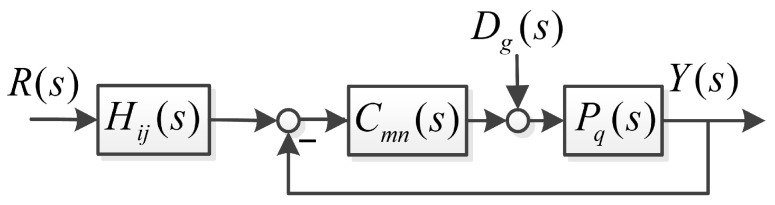
The general structure of improved linear ADRC in frequency domain.

$H_{11}\left(s\right)$, $H_{12}\left(s\right)$, $C_{11}\left(s\right)$, $C_{12}\left(s\right)$, $H_{21}\left(s\right)$, $H_{22}\left(s\right)$, $C_{21}\left(s\right)$ and $C_{22}\left(s\right)$ can be illustrated in Figure 5. In Figure 5, i∈{1,2}, j∈{1,2}, m∈{1,2}, n∈{1,2}, g∈{1,2} and q∈{1,2}.

## 4. Simulation and Discussions

To evaluate and compare the control performance of the improved ADRC and the traditional ADRC, this section presents a numerical simulation example. The liquid level control system is a typical system, and the plant is characterized by the following transfer Function (51), and the parameters of (51) can be identified by the manufacturer of liquid level systems.

(51) $$\frac{Y(s)}{U(s)}=\frac{100}{0.1s+1}$$

According to (51), the improved ADRC and the traditional ADRC are designed by referring to the Main results section.

**Figure 6:**
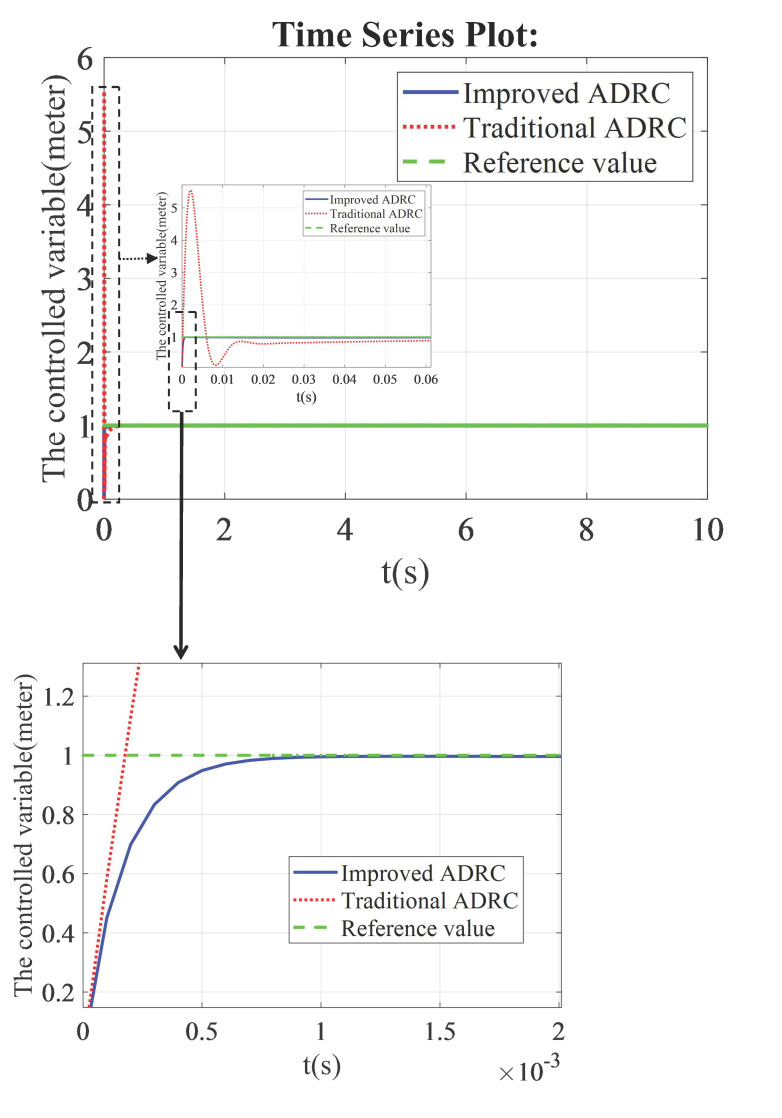
The comparison results of the improved ADRC and the traditional ADRC in the framework of the first-order ADRC.

The improved ADRC is derived from the traditional first-order ADRC. In the state feedback control law of the improved ADRC, the plant’s output value is utilized instead of relying on the output value from the extended state observer. Through simulation results analysis, it can be observed that the improved ADRC exhibits enhanced tracking performance compared to the traditional ADRC. Furthermore, Figure 6 demonstrates that the overshoots presented in traditional ADRC can be eliminated by employing the improved ADRC approach. Consequently, smooth tracking can be achieved using the improved version of ADRC. The biggest overshoot value of the traditional ADRC is about 4.5, which is about 4.5 times the set value. Tracking errors of the traditional ADRC and the improved ADRC all converge to zero. The setting time of the improved ADRC is longer than that of the traditional ADRC. The parameters of the improved ADRC and the traditional ADRC are as follows: $\beta_{11}=60$, $\beta_{12}=900$, $l_{11}=600$, ${\bar{b}}_{1}=100$.

**Figure 7:**
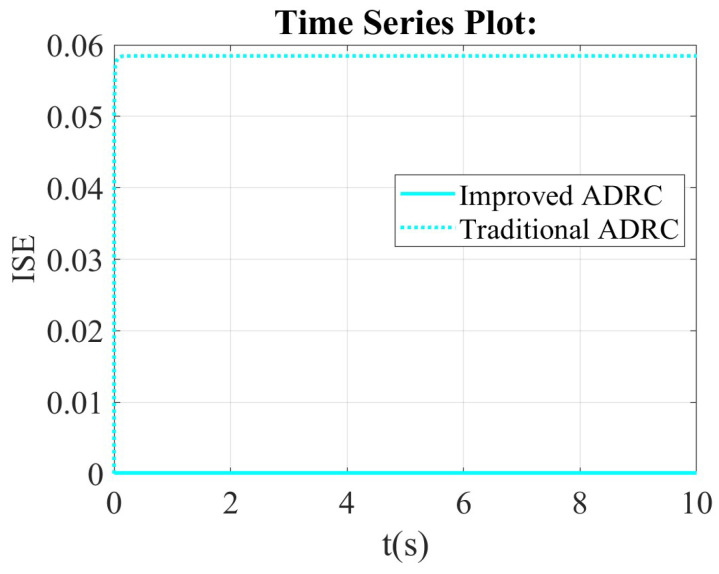
The comparison results of the improved ADRC and the traditional ADRC in the framework of the first-order ADRC in integral of squared error (ISE) scope.

From the integral of squared error (ISE) scope, the value of ISE for the improved ADRC is smaller than that of the traditional ADRC. It means that the improved ADRC reduces the maximum instantaneous error of the system. The improved ADRC is sensitive to large errors, and it can effectively suppress large overshoots and oscillations. The above description can be verified by Figure 7.

**Figure 8:**
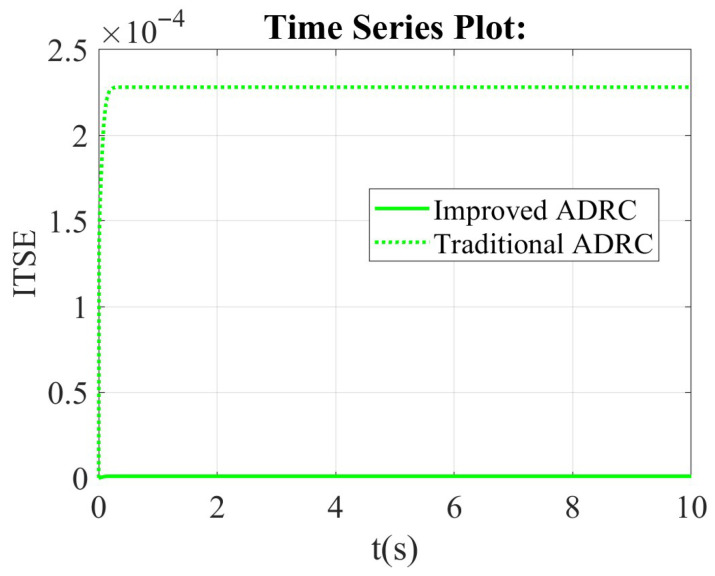
The comparison results of the improved ADRC and the traditional ADRC in the framework of the first-order ADRC in integral of time multiplied by squared error (ITSE) scope.

From the integral of time multiplied by squared error (ITSE) scope, the value of ITSE for the traditional ADRC is bigger than that of the improved ADRC in Figure 8. The value of the traditional ADRC is about zero, but that of the improved ADRC is about 0.00025.

**Figure 9:**
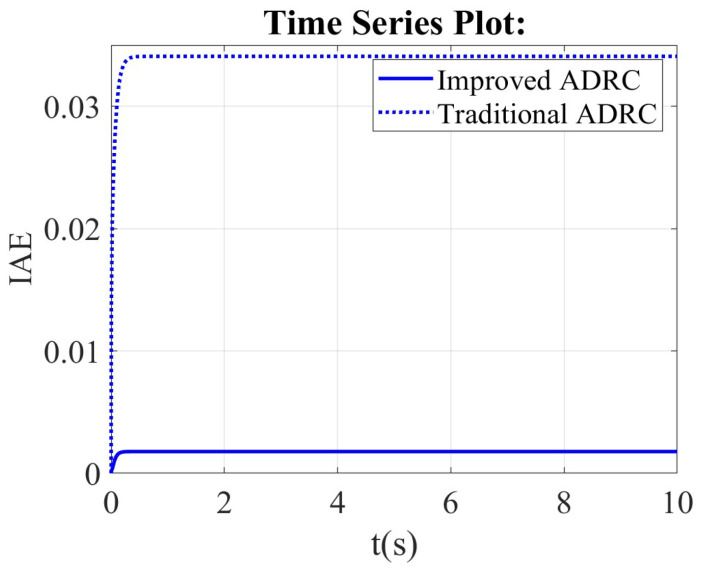
The comparison results of the improved ADRC and the traditional ADRC in the framework of the first-order ADRC in integral of absolute error (IAE) scope.

From the integral of absolute error (IAE) scope, the value of IAE for the traditional ADRC is bigger than that of the improved ADRC in Figure 9. The steady state value of the traditional ADRC is about 0.35, but the steady state value of the improved ADRC is about 0.02.

**Figure 10:**
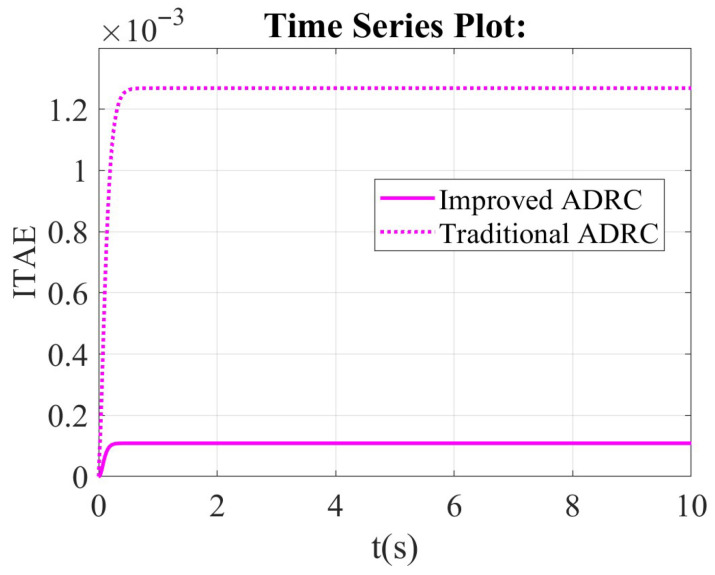
The comparison results of the improved ADRC and the traditional ADRC in the framework of the first-order ADRC in integral of time multiplied by absolute error (ITAE) scope.

From the integral of time multiplied by absolute error (ITAE) scope, the value of ITAE for the traditional ADRC is bigger than that of the improved ADRC in Figure 10. The steady state value of the traditional ADRC is about 0.00122, but the steady state value of the improved ADRC is aobut 0.0001.

DC-DC power converters are prevalent in hybrid energy vehicles and other engineering application scenarios.

**Figure 11:**
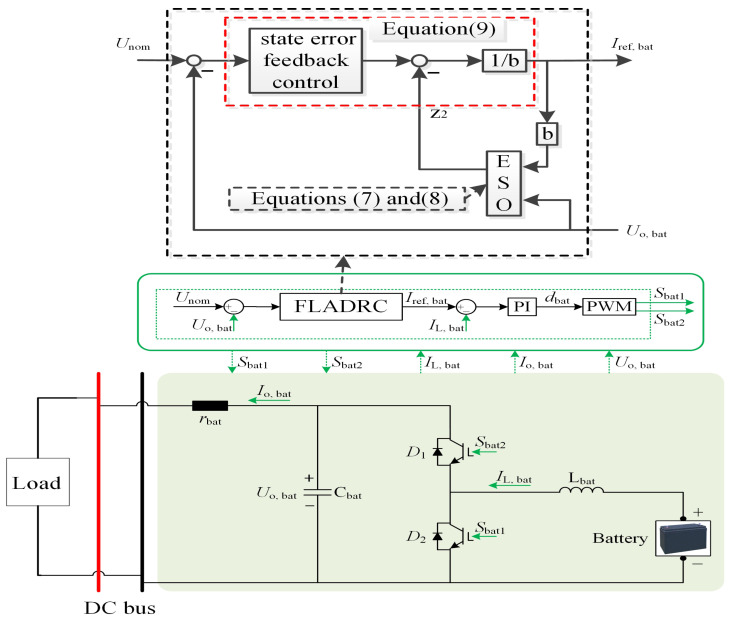
The DCDC power converter in the framework of the first-order ADRC.

Figure 11 depicts a control system for a DC bus connected to a battery and a load. The core of the system is an active disturbance rejection controller, which receives the nominal voltage ($U_{nom}$) and the battery output voltage ($U_{o,bat}$) as inputs. The ADRC generates a reference current ($I_{ref,bat}$), which is then compared to the actual battery current ($I_{L,bat}$). And the error between the $I_{ref,bat}$ and $I_{L,bat}$ is the input of the proportional–integral (PI) controller in Figure 11. The ADRC includes the traditional ADRC and the traditional ADRC. The traditional ADRC can be described by Formulas (4)–(6). The improved ADRC is illustrated by (7)–(9). The PI controller adjusts the duty cycle ($d_{bat}$) to produce pulse width modulation (PWM) signals ($S_{bat1}$ and $S_{bat2}$) that control the switching of the battery’s power electronic switches.

The DCDC section includes an inductor ($L_{bat}$), a capacitor ($C_{bat}$), and diodes ($D_{1},D_{2}$) to regulate current and voltage. The battery’s output voltage ($U_{o,bat}$) and the nominal voltage are fed back to the ADRC, forming a closed-loop control system. The load is connected to the DC bus, and the system aims to maintain stable voltage regulation by dynamically adjusting the PWM signals based on the ADRC and PI controller outputs. This configuration ensures efficient power delivery to the load while managing the battery’s charging and discharging processes.

**Figure 12:**
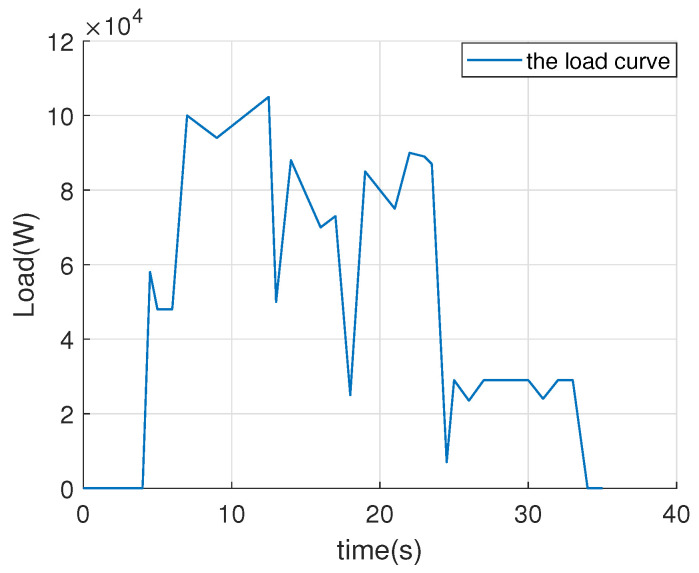
The curve of the load.

Figure 12 presents the load profile over a 35-s period, depicting the variation in power demand (in watts) with respect to time. The load remains near zero for approximately 4 s, indicating the absence of external disturbances. Subsequently, the load rapidly increases to approximately 6 × 10^4^ W within one second, reflecting a significant rise in external disturbance. Consequently, the disturbance rejection performance differs between the traditional ADRC and the improved ADRC. The improved ADRC can reduce the magnitude of DC bus voltage fluctuations more effectively than the traditional ADRC. The curve of the load decreases sharply to validate the disturbance rejection performance from 4 s to 35 s. From 0 to 4 s, the tracking performance of the traditional ADRC and the improved ADRC is compared.

**Figure 13:**
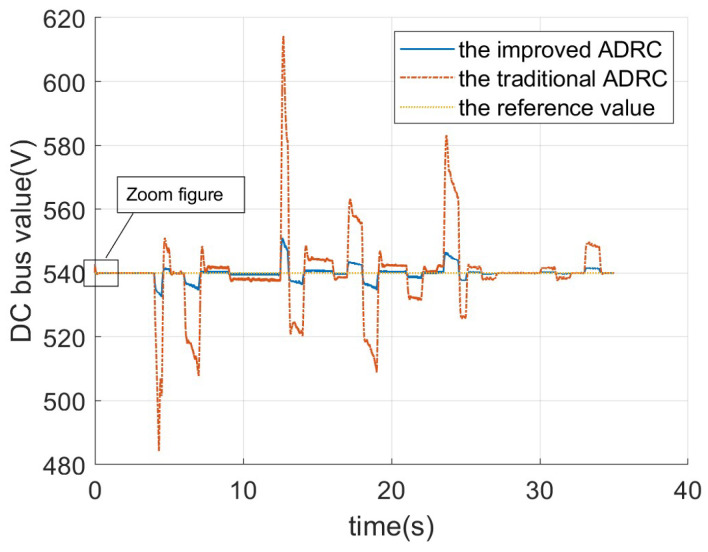
The DC voltage of DCDC power converter for different ADRCs.

Figure 13 compares the DC bus voltage values (in volts) over time (in seconds) between the improved ADRC and the traditional ADRC. The graph demonstrates that the improved ADRC achieves superior performance in maintaining a stable DC bus voltage compared to the traditional ADRC. The traditional ADRC exhibits significant fluctuations and pronounced voltage spikes, particularly during the initial phase (0–10 s) and the middle phase (15–25 s). In contrast, the improved ADRC maintains a smoother and more consistent voltage profile with reduced deviations from the nominal value. This indicates that the improved ADRC effectively mitigates voltage oscillations and enhances system robustness under dynamic operating conditions. Overall, the trend underscores the enhanced effectiveness of the improved ADRC in achieving precise voltage regulation.

**Figure 14:**
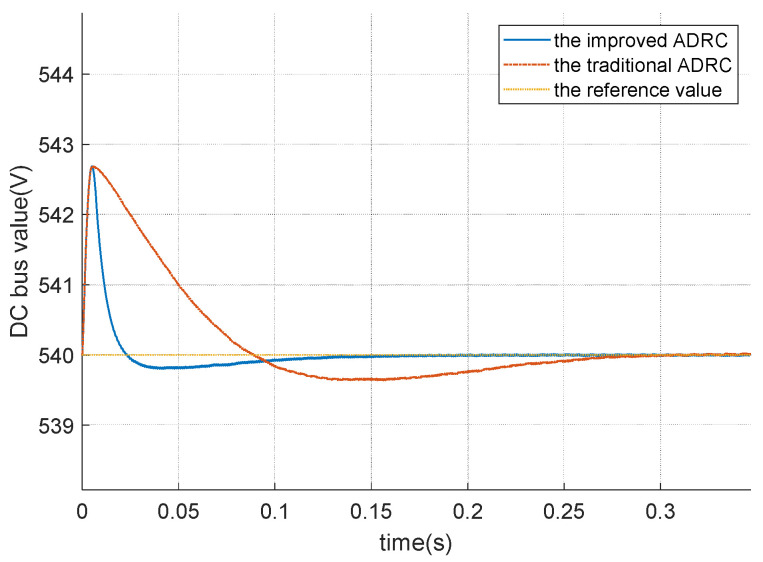
The DC voltage of DCDC power converter for different ADRCs.

The “Zoom figure” in Figure 13 is presented in Figure 14. The overshoot value of both the traditional ADRC and the improved ADRC is about 2.8 V. The time required for the traditional ADRC to fully reach the reference value is approximately 0.3 s, whereas the improved ADRC achieves the reference value in approximately 0.15 s. Therefore, the improved ADRC demonstrates superior performance in terms of response speed compared to the traditional ADRC.

**Figure 15:**
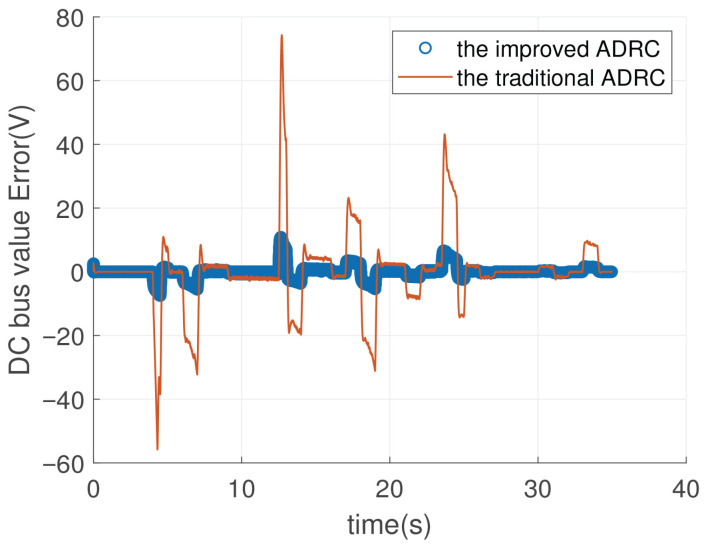
The DC voltage tracking error of DCDC power converter for different ADRCs.

**Figure 16:**
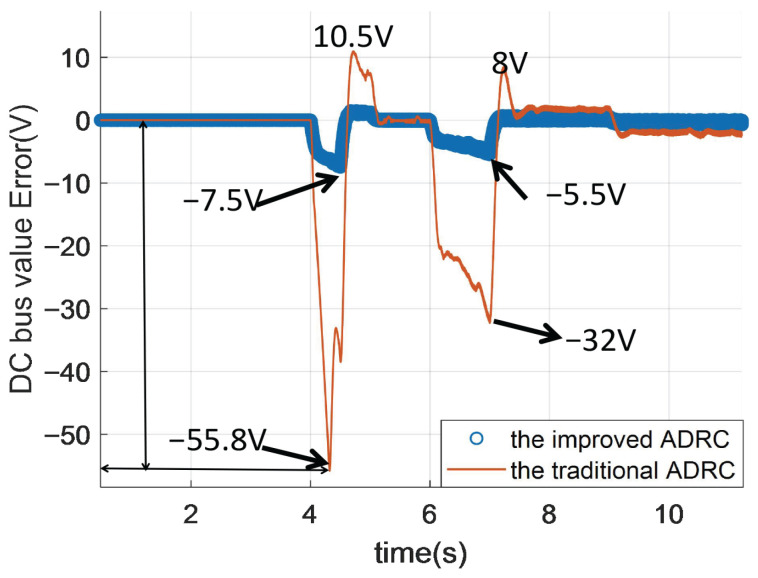
The DC voltage tracking error of DCDC power converter for different ADRCs.

**Figure 17:**
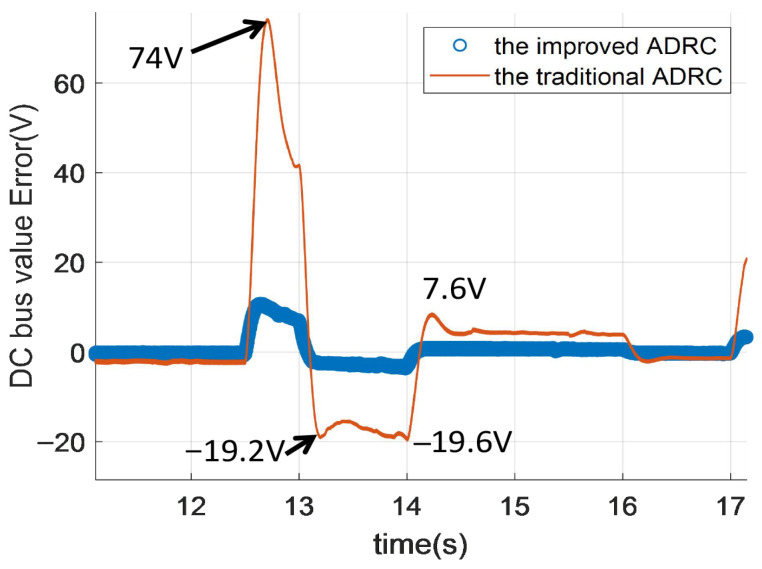
The DC voltage tracking error of DCDC power converter for different ADRCs.

**Figure 18:**
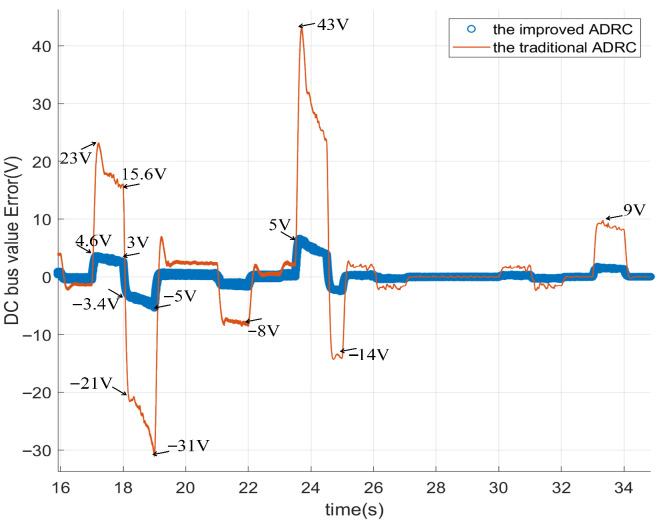
The DC voltage tracking error of DCDC power converter for different ADRCs.

Figure 15 can be obtained from the data in Figure 13. To be more specific, Figure 15 illustrates the DC bus voltage error (in volts) over time (in seconds) between the reference value and the controlled DC value for the improved ADRC and the traditional ADRC. In Figure 15, the improved ADRC value represents the difference between the reference value and the feedback value. The traditional ADRC value in Figure 15 represents the reference value minus the feedback value. In order to describe Figure 15 more clear, we give the zoom figure from Figure 15. They are named Figure 16, Figure 17 and Figure 18.

In Figure 16, from 0 to 10 s, the DC bus voltage drops for the improved ADRC are 7.5 V and 5.5 V. Thus, the biggest voltage drop is 7.5 V. However, the DC bus voltage drop values of the traditional ADRC are 55.8 V and 32 V; therefore, the biggest voltage drop is 55.8 V, which is 7.44 times the improved ADRC. As for voltage rise, the voltage rise of the improved ADRC can be neglected compared to the voltage rise of the traditional ADRC. The voltage rise value is lesser than the voltage drop for the traditional ADRC and the improved ADRC.

In Figure 17, from 10 to 17 s, the DC bus voltage drop of the improved ADRC can be ignored. However, the biggest voltage drop value of the traditional ADRC is 19.6 V. The DC bus voltage rise values of the traditional ADRC are 74 V and 7.6 V. Thus, the biggest voltage rise value is 74 V, which is 7.4 times the improved ADRC. The voltage drop value is lesser than the voltage rise value for the traditional ADRC and the improved ADRC.

In Figure 18, from 16 to 34 s, the DC bus voltage drop of the improved ADRC is 5 V. However, the biggest voltage drop of the traditional ADRC is 31 V, which is six times the improved ADRC. The largest DC bus voltage rise of the traditional ADRC is 43 V. The biggest DC bus voltage rise of the improved ADRC is 5 V, which is one-eighth of the traditional ADRC.

By summarizing the above descriptions, the graph shows that the improved ADRC demonstrates significantly smaller fluctuations and better robustness compared to the traditional ADRC. While both methods show some error variations, the traditional ADRC exhibits larger spikes and oscillations, particularly in the early stages and around 15–25 s. In contrast, the improved ADRC maintains a more consistent error close to zero, indicating superior performance in stabilizing the DC bus voltage.

## 5. Conclusions

This article establishes a mathmatical relationship between traditional linear ADRC and improved linear ADRC from the generalized PID control perspective, highlighting their distinctions in the frequency domain. Compared to the traditional ADRC, the improved ADRC incorporates differential terms and offers a novel approach to realize the generalized PID control via generalized PID interpretation. And to be more specific, the improved ADRC is a new way to realize the generalized PID control by three tuned parameters; the number of parameters of the improved ADRC is fewer than that of the generalized PID control. From the time domain, numerical simulation results demonstrate that improved ADRC exhibits superior control performance by eliminating overshoot during set value tracking processes compared to the traditional ADRC. To be more specific, the time to fully reach the reference value of the traditional ADRC is about 0.3 s, and the the time to fully reach the reference value of the improved ADRC is about 0.15 s. Thus, the improved ADRC is better than the traditional ADRC form the perspective of the time to fully reach the reference value.

From the disturbance rejection simulations in direct current to direct current converter (DCDC), the improved ADRC can achieve better disturbance rejection performance than the traditional ADRC.

The graph shows that the improved ADRC demonstrates significantly smaller fluctuations and better robustness compared to the traditional ADRC. While both methods show some error variations, the traditional ADRC exhibits larger spikes and oscillations, particularly in the early stages and around 15–25 s. In contrast, the improved ADRC maintains a more consistent error close to zero, indicating superior performance in stabilizing the DC bus voltage.

For future work, we plan to explore optimal parameter tuning for the improved ADRC, traditional ADRC, PI, PID, and PIDD^2^ controllers.

## Data Availability

The data presented in this study are available on request from the corresponding author. The data are not publicly available due to project data restriction.
